# The Role of Shared Decision-Making in Personalised Medicine: Opening the Debate

**DOI:** 10.3390/ph15020215

**Published:** 2022-02-10

**Authors:** Hector Guadalajara, Olatz Lopez-Fernandez, Miguel León Arellano, Víctor Domínguez-Prieto, Cristina Caramés, Damian Garcia-Olmo

**Affiliations:** 1 Department of General and Digestive Surgery, Fundación Jimenez Díaz University Hospital, Avda. Reyes Católicos, 2, 28040 Madrid, Spain; miguel.leon@quironsalud.es (M.L.A.); victor.dominguez@quironsalud.es (V.D.-P.); or damian.garcia@uam.es (D.G.-O.); 2 Department of Surgery, Faculty of Medicine, Universidad Autónoma de Madrid, C. Arzobispo Morcillo, 4, 28029 Madrid, Spain; 3 Department of Medical Oncology, Fundación Jimenez Díaz University Hospital, 28040 Madrid, Spain; ccarames@fjd.es

**Keywords:** shared decision-making, personalised precision medicine, healthcare, oncology, biological component, psychological component, ethics, COVID-19

## Abstract

Surgeons and cancer patients are starting to open the debate on how personalised medicine could use shared decision-making (SDM) to balance the personal and clinical components and thus improve the quality and value of care. Personalised precision medicine (PPM) has traditionally focused on the use of genomic information when prescribing treatments, which are usually pharmaceutical. However, the knowledge base is considerably scarcer in terms of how clinicians can individualise the information they provide patients about the consequences of different treatments, and in doing so involve them in the decision-making process. To achieve this, the ethical implications of SDM must be addressed from both sides. This paper explores the medical characteristics, the SDM implications in severe and fragile patients, potential risks, and observed benefits within this healthcare approach through four clinical cases. Findings shed light on current needs for clinician and patient training and tools related to SDM in PPM, and also remarks on the way in which this shift in healthcare settings is taking place to include the human component together with the biological and technological advances when designing care processes in colorectal cancer.

## 1. Introduction

Within personalised precision medicine (PPM), clinical decision-making is defined as the use of genomic information in treatment prescription, particularly drug therapy. PPM usually involves the need to generate information on health outcomes based on an evaluation of the biological component. Similar initiatives have allowed the updating of guidelines, protocols, and clinical decision aid tools addressed for the clinicians. These resources are used to better inform patients about the implications of molecular information. However, the incorporation of shared decision-making (SDM) and patient decision aid (PtDA) tools may be a key tool [1] and one that is insufficiently studied within PPM [2].

SDM facilitates the individualisation of communication between clinicians and patients to co-manage clinical information, and enhance patients’ access treatment options with principles of equity. Patients have equal rights to be fully informed about their health process, considering all alternatives and whenever they wish to share their willingness regarding the option treatments, which can mean the requirement to be equally educated to understand the clinical situation and the possible options. When necessary, PtDA tools with human support can be a great help. Theoretically, SDM in PPM extends beyond the biological component and can integrate other dimensions such as the social, psychological and personal values of patients through the educational and communicative aspects teaching them how to negotiate their treatment [2]. At the same time, PtDA tools enable clinicians to provide patients with more timely information on their condition, thereby allowing clinicians to learn about patients’ personal positions (e.g., priorities, preferences, values) regarding possible outcomes or side effects [1].

The aim of SDM is to support consensus- and evidence-based treatment selection that integrates information from different sources (e.g., biological, clinical, psychological, human) (e.g., treatment “a la carte”), specifically when there is uncertainty as to the treatment options available due to the severity of the clinical case, such as in certain oncology treatments in which no option is initially better than the others. In other words, when the clinical and personal (or well-being) components can be balanced adding patients’ preferences through an SDM approach [3].

Indeed, precision medicine usually refers to strategies outside of genomics, which include individual variability stemming from the patients’ environment and lifestyles. However, these psychosocial factors are not usually incorporated in this field of medicine, which means that the psychological, social, cultural, and economic characteristics of patients have not yet been included in evidence-based medicine [2,4]. These factors are what SDM targets to enhance and improve PPM.

In oncology and specifically in colorectal cancer (CRC) among other related diseases, the scientific literature has shown that SDM seems to work better for PPM. The reason is probably due to the interest of patients in obtaining better information about their treatment options, and the motivation from the clinicians to learn the opinion of their patients [5]. Physicians also need patients’ support in selecting a treatment, as not all decisions are part of the clinician remit [6]. The findings are promising, since trained clinicians have been able to not only agree on the treatment by partnering with patients, but have also reduced patient suffering (emotional distress) [2].

It is important to note that, for instance, for colon cancer, the set of patient-centred outcome measures is defined by the international consortium for health outcomes measurement (ICHOM [7]). It includes standard sets of outcomes connected to major treatment approaches for CRC developed through an interdisciplinary, international group of leading clinicians, measurement experts, and patient representatives. The ICHOM has found what matters to people with CRC between diagnosis and treatment selection, taking into account all dimensions related to controlling the disease, disutility of care (e.g., presence of stoma, acute complications of the treatment), degree of health (i.e., pain, depression, gastrointestinal symptoms, sexual dysfunction), and quality of death (i.e., hospital admission at the end of life, place of death).

The dimensions established by the ICHOM together with other patients’ preferences contemplate aspects from overall survival to how the consequences of treatment can influence the sexual, psychological, emotional, social, or symptomatic realms of life. In the SDM process, therefore, when deciding the best treatment for a patient, these dimensions, and probably others (e.g., age, gender, education, the influence of others, access to care or communication with the health-care provider [8,9,10]), should be taken into account. In concrete, the clinical component of the disease can be put together with the individual part of each patient. Thus, clinicians and patients can individualise and co-manage disease, creating a unique approach that balances both components.

However, a recent review on communication in decision aids for CRC [9] has found a lack of personalised treatment information. Current standards, which are the first step towards personalisation of treatment involving these cancer patients’ wishes and values, remain quite variable regarding the communication quality strategies from both sides (physicians and patients [10]) used in clinical practice.

Furthermore, there are other elements to consider in the link between SDM and PPM. For instance, genetic diagnosis has opened a path in modern medicine and requires communication with patients, because as genetic technologies are developed and the volume and complexity of data increases, new skills and strategies for service delivery and tests (e.g., liquid biopsy, massive sequencing) must be developed. Tests are sometimes not included in the service portfolios of hospitals or are not covered by insurers and must be paid for by other means; this is another reason why the patient must be aware of the cost of a particular procedure before arriving at a joint decision with their physician, recently coined as the “value of knowing” [11].

Since 1975, when the American Society for Human Genetics (ASHG) defined genetic counselling as a communication process that addresses the human problems associated with the risk of a genetic disorder in a family, SDM has slowly been developed through PPM. In 2006, the National Society of Genetic Counselors included the process of helping people understand and adapt to the medical, psychological, and family implications of genetic contributions to disease, promoting informed and shared choices (i.e., psychosocial adjustment). The National Institute for Health and Care Excellence (NICE) has published a list of recommendations when applying SDM that emphasises the need to establish this healthcare approach, train the clinicians, and provide them with PtDA tools that aid in holistic individualisation of treatment [12].

The present brief report aims to describe and discuss four case studies on the application of SDM as an approach to PPM, shedding light on the ethical issues regarding the variability of options that can emerge when surgeons, patients and patients’ family members co-manage care. These cases were managed within the Oncohealth Institute and the General Surgery and Digestive System Unit at University Hospital Fundación Jiménez Díaz (UH FJD) in 2020–2021.

## 2. Results and Discussion

Four patients received care in accordance with the SDM approach within PPM. The main personal and clinical characteristics of these patients at the moment of enrolment are summarised as follows (see Table 1 and Table 2).

### 2.1. Clinical Cases

#### 2.1.1. Patient 1

A 60-year-old male patient diagnosed in 2007 with a rectal adenocarcinoma for which he underwent low anterior resection of the rectum. The postoperative course was complicated due to a chronic anastomotic fistula, which required different procedures (i.e., Hartmann procedure, colostomy reversal with protective ileostomy, and ileostomy closure). After closure of the ileostomy, the patient developed a pelvic abscess that eventually required abdominoperineal resection after various admissions and surgical debridement.

In 2020, the patient was diagnosed with a metachronic right colon adenocarcinoma, so a right colectomy was performed. The postoperative course was again complicated due to an anastomotic leakage and an enterocutaneous fistula that required negative pressure therapy and prolonged admission until definitive surgery.

SDM was applied between the second diagnosis and the treatment selection in 2020. This patient had a long history of cancer. In his words, the stoma he had from the first oncologic intervention was what taught him about his health condition, although it occurred during a traumatic experience. After the second diagnosis, SDM was applied to begin to weigh up the options and possible consequences of each treatment between the patient (and a daughter) and the surgeon. Among the possible treatment options, the most invasive surgical option was initially selected after considering the opinion of a hospital tumour board, although the patient was initially in favour of organ preservation to preserve a segment of his colon. The patient was assuming the consequence was a second anastomosis, and he thought it was needed hence more leakage risks and tumour recurrences. In other words, he perceived the surgery as more severe than it really was.

Interestingly, the concerns of the patient were not about what treatment option to select, which is the aim of SDM [1]. His concerns were more related to a lack of awareness of the possible outcomes of the treatment options despite the previous information reported. In his previous experience, after the surgery, he woke up and observed the stoma with mixed emotions; he referred to it such “a bag on”, and expressed this was the episode he lamented more. He felt he lacked knowledge of “stoma” as a concept and believed he likely needed not only physician-provided information, but also visual depictions of stomas through images, videos, and something else (e.g., a nurse explanation of what a stoma is). He felt alone in the first postoperative process in which the stoma was already part of him. Indeed, he mentioned the need to include his family in this process of learning about the treatment options and possible consequences, as he was accustomed to co-managing his health with his family.

To include carers and family members in the SDM has been highlighted by recent studies [13,14,15], in which generally it is considered that caregivers should be involved in the patient–physician dyad. However, benefits and risks are emerging: on the one hand, they allow for a better understanding of the patients’ reasons for selecting a particular treatment option, but on the other hand, excessive caregiver involvement may create a risk of over-stepping patients’ wishes [13,14]. Similarly, recent findings with cancer patients in which SDM has been applied are highlighting physician trust, family support, and knowing the side effects of the treatment options as key factors [15]. In this first case, the patient clearly stated the need to learn about the possible side effects to avoid traumatic experiences.

In synthesis, with this first patient, the surgeon explored SDM to make the patient more aware of the treatment options and possible outcomes in this second diagnosis of adenocarcinoma. However, the postoperative course had other complications, although for reasons other than the first diagnosis, which led to a long recovery process in the hospital. From a clinician perspective, this first case reflects how even managing SDM to communicate options and jointly select a treatment may not be enough to prepare and educate the patient to face long postoperative complications.

#### 2.1.2. Patient 2

A 72-year-old male patient with a history of pelvic radiotherapy due to a prostate adenocarcinoma was diagnosed with a T3N0 low rectal adenocarcinoma.

Neoadjuvant therapy consisting of chemotherapy without radiotherapy was indicated. After neoadjuvant therapy, robotic intersphinteric resection of the rectum with terminal colostomy was performed due to an incomplete clinical response of the tumour.

Similar to the first case, the second patient also had a history of cancer. SDM was applied after the second diagnosis (i.e., rectal adenocarcinoma) and the non-surgical treatment option (i.e., neoadjuvant therapy with chemotherapy) failed to produce the desired outcome. However, as the patient was refractory to non-surgical treatment, a decision was required as to the type of surgery to perform. The surgeon applied SDM to offer two surgical treatment options: a medium-level surgery and a severe-aggressive surgery, the outcomes of which included a temporary or permanent stoma. The discussion took place with the patient and his wife. After discussing the elements related to both types of surgery, the patient agreed the surgeon should decide intraoperatively based on their observations, and the patient accepted the potential outcome of severe surgery.

However, after this initial deliberation, the patient stated that maintaining mobility was of utmost importance, as he wished to remain independent and continue to take walks and other activities of daily living. He added that the stoma would not pose a problem if he knew how to manage it. Thus, although theoretically the first outcome was to defer to the decision of the surgeon, the patient added information regarding this priority for his and his family’s quality of life. This helpful information, together with what the surgeon observed in the OR, led to the decision to perform more aggressive surgery, which resulted in a permanent stoma, which the patient received positively. There were no postoperative complications, unlike the first case. However, there was a history of cancer and active family support. The outcome was positive from the patient’s and clinicians’ points of view.

This second case evidences enhanced communication between the patient and clinicians and showed the relevance of a good connection in which unexpected messages should be facilitated and considered until the last moment before starting the treatment. As stated in the SDM literature, patient experiential evidence suggests the need to carefully balance standardized approaches and respect diversity [16]. At present, we have several models, protocols, and PTDA tools with which to exchange information. However, providing the human conditions to connect with patients’ views was more critical than any resource. It seems a more refined physician assessment of patients’ information needs and the ability to elicit patients’ preferences can be a crucial skill when applying SDM in oncology. Patients value physicians’ interpersonal skills (i.e., compassion and commitment, time spent, and listening ability) [17]. Nevertheless, physicians also need support and training to activate these soft skills when applying SDM.

Indeed, during the COVID-19 pandemic, physicians should be cautious due to the mental and emotional strain imposed by consecutive restrictions for them and their patients, e.g., restrictions in the hospital, such as family visits being forbidden during particularly severe moments in the pandemic. The situation makes them keep on track with the clinical evolution and emphasises the need to monitor patients’ well-being to preserve their mental health (e.g., patients’ distress), although the uncertainty that characterises the times of the pandemic requires mental health care for physicians [18]. In this case, SDM can facilitate continuous observation from both agents (surgeons and cancer patients) to ensure positive preoperative and postoperative phases, or even face the risk of the patients’ fear of COVID-19 infection.

#### 2.1.3. Patient 3

A 79-year-old female patient was diagnosed with a perforated cecal adenocarcinoma with an intraabdominal abscess. Right colectomy was proposed, and the patient initially accepted surgical treatment.

However, during the diagnostic process, she was also diagnosed with an acute myeloblastic leukaemia; fully aware of her diagnosis and capable of independent decision making, the patient changed her opinion and refused surgical treatment, prioritising palliative care.

This third case raised a challenge for the surgeon team in their application of SDM after the second diagnosis. Because of the second diagnosis, the patient was fragile and changed her decision regarding the treatment option (i.e., from a severe surgery to non-surgery). This negatively affected the family members, who took action to convince the patient to reconsider, as the decision not to undergo intervention, in this case, was likely to lead to death.

The situation was generated by the application of SDM. However, in one week, several successive decisions were made as the diagnosis changed. This ‘decision dance’ was stressful for the patient and family members due to the difficulty of accepting the first decision of the patient in light of the possible outcome. From the perspective of the care team, this involved tasks such as reserving the OR and then cancelling the reservation, among other clerical actions. More importantly, the surgeon who managed the SDM also bore the care burden these situations can generate in cases as complex as this third case, which was aggravated by the COVID-19 protocols in place (e.g., the family found it vexing to abide by the pandemic protocol and not visit the patient).

Compared to the previous two cases, this was the most stressful case from all sides. The severity was also higher, and the patient was more vulnerable due to fragility, although the patient did have family care. The SDM approach, therefore, is not fully developed yet and cannot be applied to complex situations such as this one, in which more psychological attention and care planning may be required, such as in palliative care (i.e., to align end-of-life care with patients’ values across different healthcare systems [19]). Indeed, the scarce studies that have applied SDM to these critical scenarios have observed that when including caregivers, most prefer to avoid discussing the patient’s foreseeable death. Prolonging life was a motivating factor for the treatment to the detriment of the quality of life or the patient’s decision. It probably requires education and politeness to keep a respectful relationship, close involvement, and open communication with healthcare professionals in the palliative scenario [20].

Similarly significant, in this case, is the fact that the situation was most likely aggravated by the restrictions mandated as part of the first lockdown in Madrid (Spain). Nonetheless, although CRC management in pandemic times now follows special guidance for delivering care (e.g., European Society for Medical Oncology), the first extended and restricted lockdowns decreased surveillance and advance-care planning worldwide [18,21]. To prevent these consequences, one key factor involves precisely improving the communication strategy to deal with diagnosis and treatment backlogs by linking the clinical and personal components [21], in which SDM within PMM offers a solution still in progress due to complexities such as those highlighted in the first three cases.

#### 2.1.4. Patient 4

An 88-year-old female patient was diagnosed with colonic adenocarcinoma located 15 cm from the anal verge. She was assessed by a geriatrist in order to evaluate her functional status.

After that, laparoscopic low anterior resection of the rectum was performed. The procedure and post-operative course were uneventful.

This fourth case raised a challenge for the surgical team in the application SDM regarding the difficulty of agreeing on a treatment option with the patient. The patient was an older person who initially agreed to make a joint decision with the surgeon. When weighing up the options, she preferred to leave the decision to a family member (i.e., her son) or the surgeon. SDM was initially undertaken between the family member and the surgeon. The former wished to solve the problem when selecting a treatment and the latter explained the treatment options. However, the patient and the family member refuse to have the right to decide with the surgeon (i.e., non-SDM), as they believed the surgeon’s decision should be followed. The surgeon explained, from his perspective, the best option was not to perform surgery, which was not the patient’s or the family member’s preferred course of action. Thus, they decided to apply SDM again to select one of the options, but only one which included surgery in agreement with the surgeon.

Theoretically, SDM requires agreement from both parties, but the literature on SDM has not yet developed this cycle of applying SDM or not during the SDM process (as it is usually voluntary and is led by patients). In this last case, the difficulty emerged when the family member decided the surgeon would select a treatment option, and the physician did not suggest what they expected. The patient and family member counter reacted and finally started the actual SDM deliberation to reach a consensus on treatment. The risk of this situation should be highlighted in the literature (i.e., non-linearity of the SDM phases, free will vs. manipulation). Clinicians may need resources and strategies to facilitate knowledge to the patients about the SDM and treatment options in order to jointly agree with them on a treatment in minimum terms with no manipulation from any side. It is suggested the role is performed to act as an ally rather than a manipulator, as SDM will request from the clinicians to manage reactive patients’ attitudes while acting receptively from both sides [22].

#### 2.1.5. Integrating Shared Decision-Making Cases within Personalized Precision Medicine

This study integrates the personal issues affecting therapeutic adjustment in SDM towards a PPM. The diagnosis is based on genomic information and medical prescription, which goes further than pharmaceutical treatments in CRC, covering the psychosocial component intricate when jointly selecting a treatment with the patient and family members.

According to Juengst et al. [23], the change from personalised to precision medicine from the traditional ethos of clinical genetics generates ethical concerns (i.e., from clinical interpretation to responsibility coaching, informative censoring to involuntary genetic testing and disclosure). They argued about how to deal with the predictive and uncertainties of the decisions that geneticists and genetic counsellors help their patients make. The patient-centred approach has risen this century with ‘patient autonomy’, and precisely it seems to be the weakness of ‘personalised genomic medicine’—patient empowerment in terms of the individualisation of care. Meanwhile, in bioethics, its paradigms move towards relational autonomy, solidarity, and more nuanced understandings of SDM.

The description and discussion of the four cases, from the clinical and healthcare perspectives, show that the theoretical implementation of SDM within PPM is not so clearly established as it initially seemed based on a current state of the art [2,3,4,7,8,9,10,11]. Nevertheless, although societies and international consortiums are starting to ask about patients’ involvement in their health decisions [7,12,23,24], more clinical and psychological research is needed to learn how to manage the personal component of this humanistic approach which cancer patients are requesting [13,14,15,16,17,19,20,22]. In this study, the emerging scenarios are probably due to the individual variability, the critical time in which patients and surgeons managed this approach (i.e., the first waves of the COVID-19 pandemic [21]), the ethical considerations, and the lack of maturity in current evidence-based medicine regarding SDM medical practice.

## 3. Materials and Methods

A retrospective narrative review was performed from four clinical cases, selecting patients who voluntarily underwent SDM within PPM in CRC surgeries in the FJD (Spain) between November 2020 and July 2021. The study was reported after obtaining approval from the local institutional review board committee.

## 4. Conclusions

In conclusion, the process of bringing clinicians and patients closer together to agree on the healing path by using both molecular and personal evidence has shown a diversity of outcomes usually not reported in the SDM and PPM literature. The present study offers an analysis of four clinical cases and discusses the relevance of preparing and educating patients to face complex decisions in CRC and possible long postoperative phases through consensual decisions between them and their physicians. At the same time, it has highlighted the need to train and support physicians in communication strategies to know what is vital for patients’ daily lives, and abilities to face reactions is crucial to address SDM in a dynamic PPM setting. Furthermore, more development regarding decision aids linked to communication strategies from both sides can support some of the deficiencies detected in the literature and shown alongside the cases to cover the dimensions to consider from patients’ perspectives regarding the best treatment for them. In the near future, other considerations such as making an effort to equally monitor the clinical and personal components can probably be facilitated by the aids and strategies to develop within the healthcare systems which will apply the SDM in the PPM (e.g., to design and develop care processes centred in the persons diagnosed with a CRC to treat the tumour while preserving mental health and wellbeing). Thus, surgeons’ human skills are critical to successfully activating SDM processes, apart from the willingness to collaborate from the patients’ side. Similarly, families seem to be relevant factors in the SDM for managing CRC within PPM but should be involved with caution, and all agents should act as allies to work in partnership with lack of manipulation. Finally, emotional distress in palliative care situations can emerge from SDM in fragile patients, which is a pending element to support patients (and families) and clinicians who manage the emotional consequent outcomes.

## Figures and Tables

**Table 1:** Patient characteristics related to the SDM in the PPM approach.

Patient ID	Gender	Age	Family Member	Diagnostic for SDM	Possible Surgical Treatments	Physician Communication Strategy	Patient Communication Style	SDM Communication Strategy	Main Patient Concerns
Patient 1	Male	60	Daughter	Metachronic right colon adenocarcinoma	Medium-level surgery or severe-aggressive surgery	Explain CRC Analyse test results Describe options and their implications Scheduling strategiesGive advice to improve symptoms Jointly agree on an option with the patient Monitor him	Assertive style of symptom communication (e.g., seriousness of the symptoms, family history, asking questions)	Analysing the options and possible consequences with the patient and family member	Unawareness of the possible outcomes of the treatment options
Patient 2	Male	72	Wife	Low rectal adenocarcinoma	Medium-level surgery or severe-aggressive surgery	Explain CRC Analyse test results Describe options and their implications Scheduling strategies Give advice to improve symptoms Jointly agree on an option with the patient Monitor him	Assertive style of symptom communication	Discussion between the surgeon, the patient and his wife	Maintaining mobility to remain independent
Patient 3	Female	79 *	Son and his family	Cecal adenocarcinoma with an intraabdominal abscess, and an acute myeloblastic leukaemia	Severe surgery or no surgery (palliative treatment)	Explain CRC Analyse test results Describe options and their implications Jointly agree on an option with the patient and family Refer patient to a palliative-care specialist	Less assertive communication style (e.g., too sick to say very much)	Discussion between the surgeon, the patient, and the family with frequent changes made to the decision (‘decision dance’)	To agree with the family decision
Patient 4	Female	88 *	Son	Colonic adenocarcinoma	Severe surgery or no surgery (palliative treatment)	Explain CRC Analyse test results Describe options and their implications Jointly agree on an option with the patient Referred patient to a palliative-care specialist	Less assertive communication style (e.g., mentioning symptoms while discussing other medical issues)	Analysing the options and possible consequences with the family member	To be treated surgically following agreement with the family member

* These patients are considered fragile due to their advanced age and diseases.

**Table 2:** Patient clinical characteristics.

Patient ID	Gender	Age	Surgery	Oncological Treatments	Surgical Specimen	TNM ed. AJCC UICC 2018	Immunohistochemistry	Genetic Information	Current Status
Patient 1	Male	60	Partial colectomy without stomas	Due to severe complications, the cannot undergo oncological therapy	Colloid adenocarcinoma No venous, lymphatic, or perineural involvement Free margins	pT3N0 (0 of 35)	Mlh1 and PMS2: loss of expression MSH23 and MSH6: expression preserved CDX2 Positive	Kit Cobas BRAF mutation test IVD: BRAF WT PCR detects high microsatellite instability	Oncologic disease-free, Home parenteral nutrition, bilateral hydronephrosis
Patient 2	Male	72	Rectal anterior resection with definitive stoma	8 cycles of FOLFOX before surgery (total neoadjuvant therapy) without radiotherapy. Patient treated with radiotherapy in 2015 due to a prostate cancer.	No venous, lymphatic, or perineural involvement Radial margin affected	ypT3N0 (0 of 12)	Mlh1 and PMS2, MSH23 and MSH6: expression preserved CDX2 Positive	NA	Biochemical prostate cancer relapse. No rectal cancer relapse.
Patient 3 *	Female	79 *	No surgery	No treatment	NA	NA	NA	NA	Exitus
Patient 4 *	Female	88 *	Sigmoidectomy with anastomosis	No treatment	Venous and lymphatic involvement	pT2N0 (0 of 16)	Mlh1 and PMS2, MSH23 and MSH6: expression preserved CDX2 Positive	NA	Institutionalized due to mobility deficiencies.

* These patients are considered fragile due to the older age and diseases.

## Data Availability

Data is contained within the article.
